# HOW DO PRIMARY CARE PHYSICIANS NAVIGATE MEDICAL CANNABIS DISCUSSIONS WITH OLDER ADULT PATIENTS

**DOI:** 10.1093/geroni/igae098.1241

**Published:** 2024-12-31

**Authors:** Annie Nguyen, Pearse O’Malley, Edward Osae-Oppong, Jamie Foo, Dania Abu-Baker, Lize Tibirica, Joseph Diaz, Alison Moore

**Affiliations:** University of California San Diego, La Jolla, California, United States; Tulane University School of Medicine, New Orleans, Louisiana, United States; California State University San Marcos, San Marcos, California, United States; University of California San Diego, La Jolla, California, United States; University of California San Diego, La Jolla, California, United States; University of California San Diego, La Jolla, California, United States; University of California San Diego, La Jolla, California, United States; University of California San Diego School of Medicine, San Diego, California, United States

## Abstract

We interviewed 20 physicians who provide primary care in California and have patients ages 65+ in their practice. Interviews lasted 30 minutes and followed a semi-structured guide. The majority of physicians said that older patients were most likely to use cannabis for pain and sleep issues while younger patients used it for mental health-related conditions. Older patients often seek physicians’ advice about doses, forms, and efficacy, but most physicians were unsure on how to advise patients, with many defaulting to the strategy of starting with low doses and titrating up if needed; inhalation was universally discouraged. Many physicians were unsure about the strength of evidence, because while the evidence for sleep and pain appear to be supportive, there are not enough studies and large-scale clinical trials to develop a strong opinion. All agreed that physicians would benefit from training to increase confidence and be informed when discussing cannabis with patients.

